# Circumsporozoite protein rates, blood-feeding pattern and frequency of knockdown resistance mutations in *Anopheles* spp. in two ecological zones of Mauritania

**DOI:** 10.1186/s13071-016-1543-0

**Published:** 2016-05-05

**Authors:** Khadijetou Mint Lekweiry, Mohamed Salem Ould Ahmedou Salem, Christelle Cotteaux-Lautard, Fanny Jarjaval, Adeline Marin-Jauffre, Hervé Bogreau, Leonardo Basco, Sébastien Briolant, Ali Ould Mohamed Salem Boukhary, Khyarhoum Ould Brahim, Frédéric Pagès

**Affiliations:** UR Génome et Milieux, Faculté des Sciences et Techniques, Université des Sciences, de Technologie et de Médecine, Nouveau Campus Universitaire, BP 5026, Nouakchott, Mauritania; Institut de Recherche Biomédicale des Armées, Ancienne base aérienne 217, B.P. 73, 91223 Brétigny-sur-Orge, France; Laboratoire de Parasitologie, CNR du Paludisme région Antilles-Guyane, Institut Pasteur de la Guyane, Cayenne, Cedex, France; Unité de Recherche sur les Maladies Infectieuses et Tropicales Emergentes, Unité de Recherche 198-Institut de Recherche pour le Développement, Faculté de Médecine La Timone, Aix-Marseille Université, 27 Boulevard Jean Moulin, Marseille, 13385 France; Regional office of the French Institute for Public Health Surveillance, Cire Océan Indien, Saint-Denis, Reunion Island France

**Keywords:** *Anopheles arabiensis*, *Anopheles gambiae* (*s.s.*), CSP*-*ELISA, Blood meal, *Plasmodium vivax* VK210, Mauritania

## Abstract

**Background:**

Mosquitoes belonging to *Anopheles gambiae* species complex are the main malaria vector in Mauritania but data on their vector capacities, feeding habits and insecticide susceptibility are still scanty. The objectives of this study were to fill this gap.

**Methods:**

Adult *Anopheles* spp. mosquitoes were collected using pyrethrum spray catch method from two ecological zones of Mauritania: Nouakchott (Saharan zone) and Hodh Elgharbi region (Sahelian zone). Circumsporozoite proteins (CSP) for *P. falciparum*, *P. vivax* VK210 and *P. vivax* VK247 were detected by enzyme-linked immunosorbent assay (ELISA) from the female anopheline mosquitoes. To confirm CSP-ELISA results, polymerase chain reaction (PCR) was also performed. Blood meal identification was performed in all engorged females by partial sequencing of the mitochondrial cytochrome *b* gene. Molecular assessments of pyrethroid knockdown resistance (*kdr*) and insensitive acetylcholinesterase resistance (*ace*-1) were conducted.

**Results:**

In Nouakchott, the only species of *Anopheles* identified during the survey was *Anopheles arabiensis* (356 specimens). In Hodh Elgharbi, 1016 specimens of *Anopheles* were collected, including 578 (56.9 %) *Anopheles rufipes*, 410 (40.35 %) *An. arabiensis*, 20 (1.96 %) *An. gambiae*, 5 (0.5 %) *An. pharoensis* and 3 (0.3 %) *An. funestus*. Three of 186 female *An. arabiensis* collected in Nouakchott and tested by ELISA were found positive for *Plasmodium vivax* VK210, corresponding to a sporozoite rate of 1.6 %; however PCR confirmed infection by *P. vivax* sporozoite in only one of these. In Hodh Elgharbi, no mosquito was found positive for *Plasmodium* spp. infection. There was a statistically significant difference in the percentage of human blood-fed *Anopheles* spp. between Nouakchott (58.7 %, 47 of 80 blood-engorged *An. arabiensis* females) and Hodh Elgharbi (11.1 %, 2 of 18 blood-engorged mosquitoes). Analysis of the *kdr* polymorphisms showed 48.2 % (70/145) of East African *kdr* mutation (L1014S) in Nouakchott compared to 10 % (4/40) in Hodh Elgharbi region (*P* < 0.001). Nevertheless, West African *kdr* mutation (L1014F) was found only in *An. gambiae* populations (4/40, 10 %) from Hodh Elgharbi region. No *ace-1* mutation was found in mosquito specimens from the two study zones.

**Conclusions:**

Overall, this study confirmed the autochthonous *P. vivax* malaria transmission in Nouakchott, involving *An. arabiensis* as the main vector. It also described for the first time the absence of *ace-1* mutation, the co-occurrence of both West and East African *kdr* mutation in *An. gambiae* in Mauritania, and highlighted the regional variations in the prevalence and type of *kdr* mutations.

## Background

Mauritania (15°–27°N, 5°–17°W) covers three ecological zones. The Saharan zone in the north where rainfall is scarce (< 100 mm annually) covers two-thirds of the surface area of the country. The Sahelian zone, characterised by an annual rainfall of 100–300 mm, extends through much of the southern and the southeastern parts of the country. The Sahel-Sudanese zone, located in the southernmost part of the country with an annual rainfall of 300–400 mm, includes Guidimagha and part of the Gorgol regions. There is only a short rainy season that extends from June/July to September/October, depending on the year and the region. All ecological zones support the presence of mosquitoes, including malaria vectors (*Anopheles* spp.). Malaria transmission is seasonal in Mauritania with a peak at the end and shortly after the rainy season (September to November) [1]. Most infections in the Sahelian and the Saharan zones of the country are due to *Plasmodium falciparum* and *Plasmodium vivax*, respectively [2, 3]. However, there is a lack of epidemiological data in the Sahel-Sudanese zone, and the situation of malaria in this area is possibly different from other parts of the country.

Twelve anopheline species have been reported in Mauritania [4–8] including the major malaria vectors in Africa, *Anopheles gambiae*, *An. arabiensis* and *An. funestus. Anopheles* spp. exhibit a wide range of blood meal preferences, such as humans, livestock, birds and reptiles that could change for the same species according to local conditions and/or host availability [9]. Since feeding habits (host preference, indoor or outdoor feeding and biting activity hours) are highly variable, only a local assessment can reveal pertinent data. Knowledge on the behaviours, including feedings (exo- or endophagia) and resting habits (exo- or endophily), of *Anopheles* spp. mosquitoes, as well as the detection of parasites in mosquito salivary glands, remains an integral component in understanding the transmission dynamics of malaria. These data are essential to adopt an appropriate strategy for vector control operation in an area but have to be completed by an assessment of the insecticide susceptibility of *Anopheles* spp. mosquitoes using a standardised World Health Organisation (WHO) assay and molecular detection.

Malaria transmission has been reported in all areas of Mauritania but the burden of the disease varies according to local conditions. Between 2000 and 2015 due to control interventions, the modelled estimates of malaria case incidence as well as the admission rates have fallen by more than 50 %. Nevertheless, 70 % of the population (estimated at 3,537,368) is living in a high transmission area (more than one case per 1000 inhabitants per year) and 30 % is living in a low transmission area (less than one case per 1000 inhabitants per year). In 2013, the number of confirmed malaria cases was estimated between 40,000 and 120,000 cases and the number of malaria deaths between 240 and 1500 (http://www.who.int/malaria/publications/country-profiles/profile_mrt_en.pdf). In 2012, malaria was the first cause of mortality in the southern part of the country. Although malaria prevalence is decreasing in west Africa, the success of malaria control in many countries is threatened by the spread of insecticide resistance (pyrethroids, DDT and also carbamates) as well by the behaviour modification of the main malaria vectors [10–12]. Data on the status of insecticide resistance in different malaria vectors in Mauritania are essential to contribute to the success of malaria control program and it is necessary to clarify the role of different vectors present in the country and their distribution.

The present study was carried out to describe the anopheline species present, their *P. vivax* and *P. falciparum* infection rates, their anthropophilic rates as well as the presence and frequency of Kdr and Ace-1 mutations associated with insecticide resistance in two ecological zones of Mauritania.

## Methods

### Study areas

**Fig. 1:**
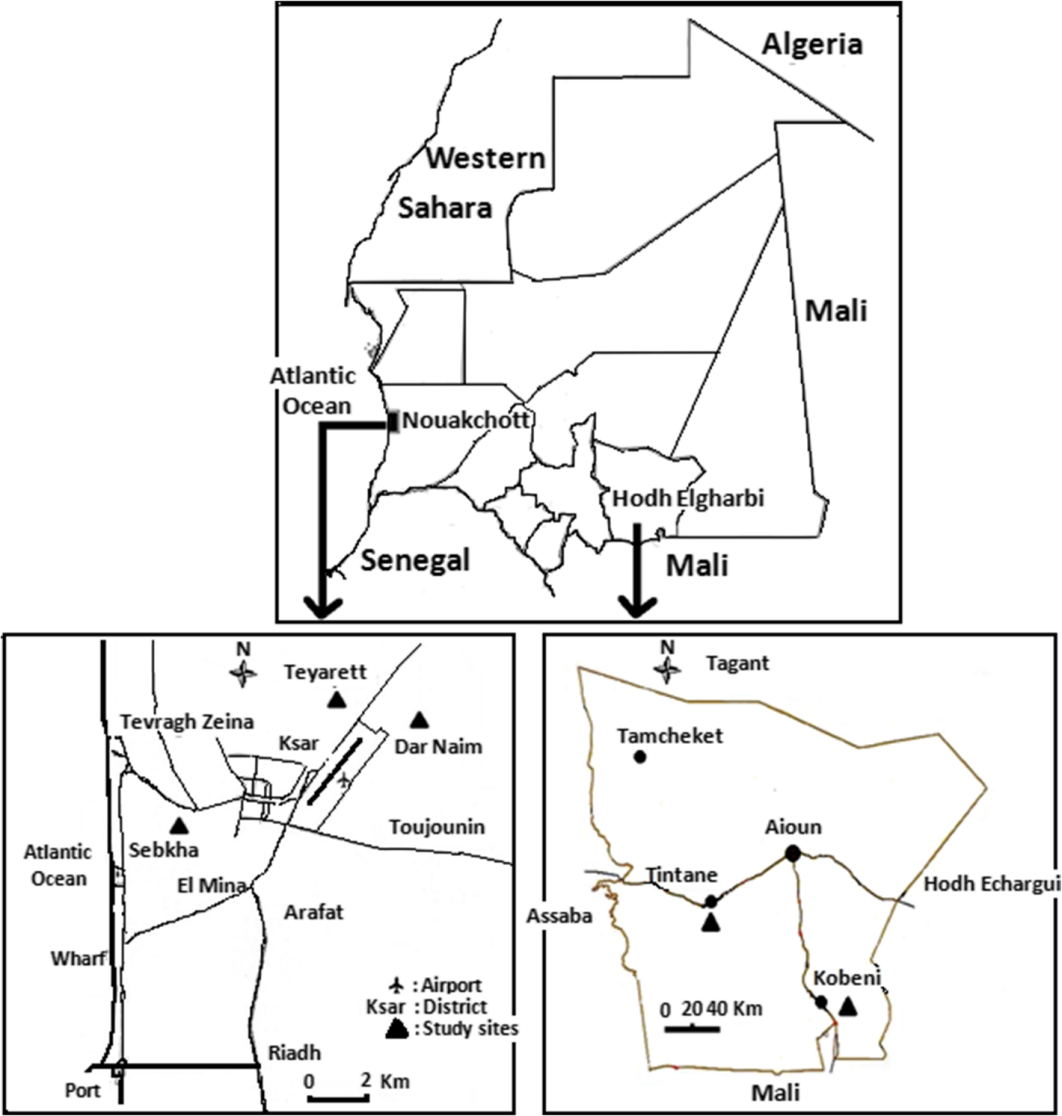
Map of Mauritania showing Hodh Elgharbi and Nouakchott and the corresponding mosquito sampling sites

The study was conducted at five sites in Mauritania: Teyarett (18°07′40″N, 15°56′21″W, altitude 4.3 m), Dar Naim (18°06′14″N, 15°55′43″W, altitude 2 m) and Sebkha (18°04′27″N, 16°00′16″W, altitude 3 m) districts in Nouakchott, the capital city of Mauritania, located in the Saharan part of the country, and Kobeni (15°49′02″N, 9°22′55″W, altitude 198 m) and Tintane (16°18′43″N, 10°10′01″W, altitude 169 m) districts in Hodh Elgharbi region situated in the Sahelian part of the country (Fig. 1) [13]. Nouakchott has a population of 834,000 (25 % of the country’s population). The climate is characterised by a short rainy season (July-September) with an average annual rainfall of 50–80 mm. The average annual temperature ranges from 20.7° to 33.3 °C, and the average relative humidity ranges from 33 to 79 %. Hodh Elgharbi region (16°13′02″N, 9°54′ 44″W) covers a surface area of 53,000 km^2^ and includes four departments (Aïoun, Kobeni, Tintane and Tamchekett). The population of Hodh Elgharbi is estimated as 294,109 inhabitants [13]. The climate in Hodh Elgharbi region is predominantly Sahelian, characterised by a long dry season lasting from October to June and a rainy season extending from July to September. The mean annual rainfall is between 200 and 300 mm, and the mean relative humidity ranges from 19 to 43 %. The mean monthly temperature ranges from 24 to 37 °C. The area is mostly made up of steppes and savanna grasslands. Human activities are dominated by livestock rearing (mainly cattle, sheep and goats) and agriculture (mainly millet).

### Mosquito sampling

Indoor resting *Anopheles* spp. mosquitoes were collected in Nouakchott from January 2009 to December 2010 involving three dry seasons (from January to June 2009, from October 2009 to June 2010 and from October to December 2010) and two rainy seasons (from July to September 2009 and from July to September 2010), and in Hodh Elgharbi region from July to October 2010 covering one rainy season, using pyrethrum spray catch (PSC). In Nouakchott, mosquito collections were performed twice monthly during the dry season (October–June) and every week during the rainy season (July–September), while in Hodh Elgharbi region, mosquito samples were collected only during the rainy season (July–September). PSCs were performed at 07:00 AM by spraying deltamethrine for 30–45 s in the room. After 10 min, dead and immobilised mosquitoes were collected.

### Mosquito identification

Mosquitoes were morphologically identified in the field according to the taxonomic keys developed by Gillies & De Meillon [14]. Female *Anopheles* spp. mosquitoes were then classified according to their feeding status as unfed, fed, half-gravid or gravid, and individually stored for subsequent analyses. Genomic DNA was extracted from the legs and wings of female mosquitoes using QIAamp® Media MDx® kit with the Biorobot® (Qiagen, Marseille, France) according to the manufacturer’s instructions. Molecular identification of cryptic species in the *Anopheles gambiae* complex (*An. coluzzii* and *An. gambiae*) was performed with species-specific PCR amplification [15].

### Circumsporozoite protein and blood meal ELISA

Enzyme-linked immunosorbent assay (ELISA) was performed to detect the circumsporozoite surface protein (CSP) of *P. falciparum* with monoclonal antibody Pf2A10-01, *P. vivax* 210 with monoclonal antibody VK-210, and *P. vivax* 247 with monoclonal antibody VK-247 in the head-thoracic portion of the mosquitoes, according to the method of Burkot et al. [16] modified by Wirtz et al. [17]. All CSP-ELISA-positive *Anopheles* were further analysed by specific nested PCR to detect *P. falciparum* and *P. vivax* infection from mosquito DNA extract as previously described [18]. The abdomens of blood-fed female *Anopheles* spp. were used to determine the origin of blood meal. Blood and tissue samples from engorged females were disrupted by mechanical homogenisation in Phosphate Buffered Saline (PBS; P3813-10PAK, Sigma Aldrich, Saint-Quentin Fallavier, France) containing 3 % bovine serum albumin (BSA). Whole blood DNA was extracted from the abdomen using the QiaAmp blood DNA mini Kit (Qiagen, Courtaboeuf, France) and eluted in 50 μl of elution buffer. The primer pair L14841 (5′-AAA AAG CTT CCA TCC AAC ATC TCA GCA TGA TGA AA-3′) and H15149 (5′-AAA CTG CAG CCC CTC AGA ATG ATA TTT GTC CTC A-3′) were used to amplify a 305 bp segment of the mitochondrial cytochrome *b* gene from the host DNA [19]. PCR products were examined by electrophoresis in a 2 % agarose gel, purified using Wizard® SV Gel kit and PCR Clean-up System (Promega, Charbonnières-les-Bains, France) and sequenced directly in cycle sequencing reactions using the primer H15149 (Big Dye, Applied Biosystems, Villebon–sur-Yvette, France,). Sequence data were used for species identification with the nucleotide - nucleotide basic alignment search tool (BLAST) in GenBank DNA sequence database (NCBI) [20].

### Knockdown resistance (*kdr*) and insensitive acetylcholinesterase (*ace-1*) gene mutations

Mosquitoes belonging to *An. gambiae* complex were tested for both west (L1014F) and east (L1014S) *kdr* mutations using *TaqMan*-q PCR assays in which two *TaqMan* real-time reactions were performed in parallel to differentiate the two resistant alleles [21]. The first reaction used primers *kdr*-F (5′-CAT TTT TCT TGG CCA CTG TAG TGA T-3′), and *kdr*-R (5′-CGA TCT TGG TCC ATG TTA ATT TGC A-3′), and probes WT (5′-CTT ACG ACT AAA TTT C-3′) labelled with VIC^TM^ at the 5′ end for the detection of the wild-type allele and FAM1 (5′-ACG ACA AAA TTT C-3′) labelled with 6-FAM^TM^ to detect L1014F allele. The second reaction used primers *kdr*-F and *kdr*-R, and probes WT and FAM2 (5′-ACG ACT GAA TTT C-3′) to detect L1014S allele. Each reaction included at least one negative control (double deionised molecular grade water). All samples and controls were amplified in triplicate. Each PCR reaction used 5 μl of mixture consisting of 2.5 ng of mosquito genomic DNA, 2.5 μl of 2× PCR Mastermix (*TaqMan* universal PCR master mix, Applied Biosystems, Villebon-sur-Yvette, France), 900 nM of each primer and 200 nM of each probe. The cycling conditions were 50 °C for 2 min for carry-over inactivation, and 95 °C for 10 min, followed by 40 cycles of 92 °C for 15 s and 60 °C for 1 min. Samples were run on an ABI PRISM 7900 HT sequence detection system in 384-well format (Applied Biosystems). The system automatically monitors the increase in VIC and FAM fluorescence intensities in real time at each cycle. The *ace-1* mutation G119S was detected in the mosquito genomic DNA by PCR according to the protocol of Weill et al. [22]. PCR products were digested with *Alu* I restriction enzyme (Promega, Charbonnières-les-Bains, France) at 37 °C for 3 h.

### Data analysis

Data were analysed with Stata 11® statistical software. Blood-feeding pattern, distribution and prevalence of *kdr* mutations in mosquito populations were compared between the study areas using Fisher’s exact test. The level of significance was fixed at *P* < 0.05.

## Results

### Species diversity in Nouakchott and Hodh Elgharbi

**Fig. 2:**
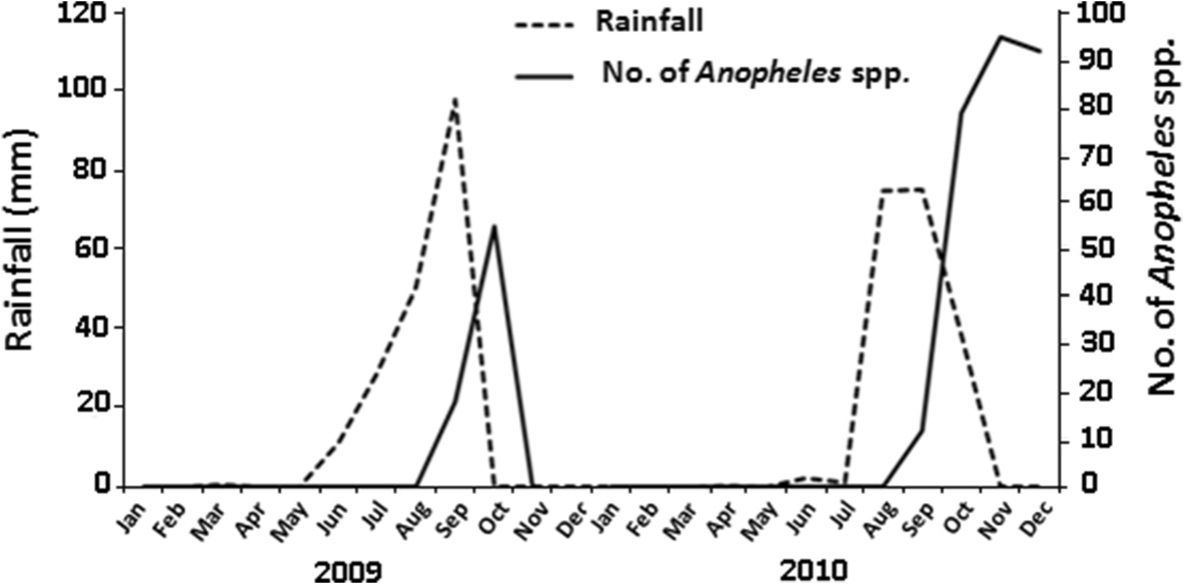
Seasonal abundance of *Anopheles* spp. collected using pyrethrum spray and rainfall in Nouakchott in 2009 and 2010

In Nouakchott, 39,304 mosquito specimens were sampled in this study among which 22,231 mosquitoes were collected in 2009 and 17,073 in 2010. From January to July 2009, October 2009 to June 2010, and from October to December 2010, corresponding to the dry season, all mosquitoes collected at rest in human dwellings were *Culex* spp. However, during and shortly after the rainy season (August to November 2009 and August to December 2010), specimens of *Anopheles* spp. were found among captured culicid fauna in Dar Naim (73 specimens in 2009 and 180 specimens in 2010) and Teyarett (none in 2009 and 103 specimens in 2010) districts. No *Anopheles* spp. specimens were captured in 2009 and in 2010 in Sebkha district. The seasonal variations of the *Anopheles* spp. population density and of the rainfall are presented in Fig. 2. All *Anopheles* spp. captured were morphologically identified as *An. gambiae* (*s.l*.), with the proportions of 0.33 % (73/22,231) in 2009 and 1.6 % (283/17,073) in 2010 (Table 1). However, molecular identification of 186 specimens revealed the presence of only *An. arabiensis* within *An. gambiae* (*s.l*.) tested specimens.

**Table 1:** Diversity of indoor resting *Anopheles* spp. collected using pyrethrum spray in Nouakchott (Saharan zone) in 2009 and 2010 and in Hodh Elgharbi region (Sahelian zone) in 2010

Study sites	*Anopheles* species				
	*An. gambiae* (*s.s.*)	*An. arabiensis*	*An. funestus*	*An. pharoensis*	*An. rufipes*
Nouakchott					
Teyarett	0	103	0	0	0
Dar Naim	0	253	0	0	0
Sebkha	0	0	0	0	0
Total	0	356	0	0	0
Hodh Elgharbi					
Kobeni	16	274	2	4	394
Tintane	4	136	1	1	184
Total	20	410	3	5	578

In Hodh Elgharbi region, catches were focused only on *Anopheles* spp. Data on other genera were not recorded. The overall species composition of the 1016 collected *Anopheles* spp. mosquitoes comprised 578 (56.9 %) *An.rufipes*, 410 (40.35 %) *An. arabiensis*, 20 (1.96 %) *An. gambiae* (*s.s.*), 5 (0.5 %), *An. pharoensis*, and 3 (0.3 %) *An. funestus*.

### Blood-feeding patterns and sporozoite rates

**Fig. 3:**
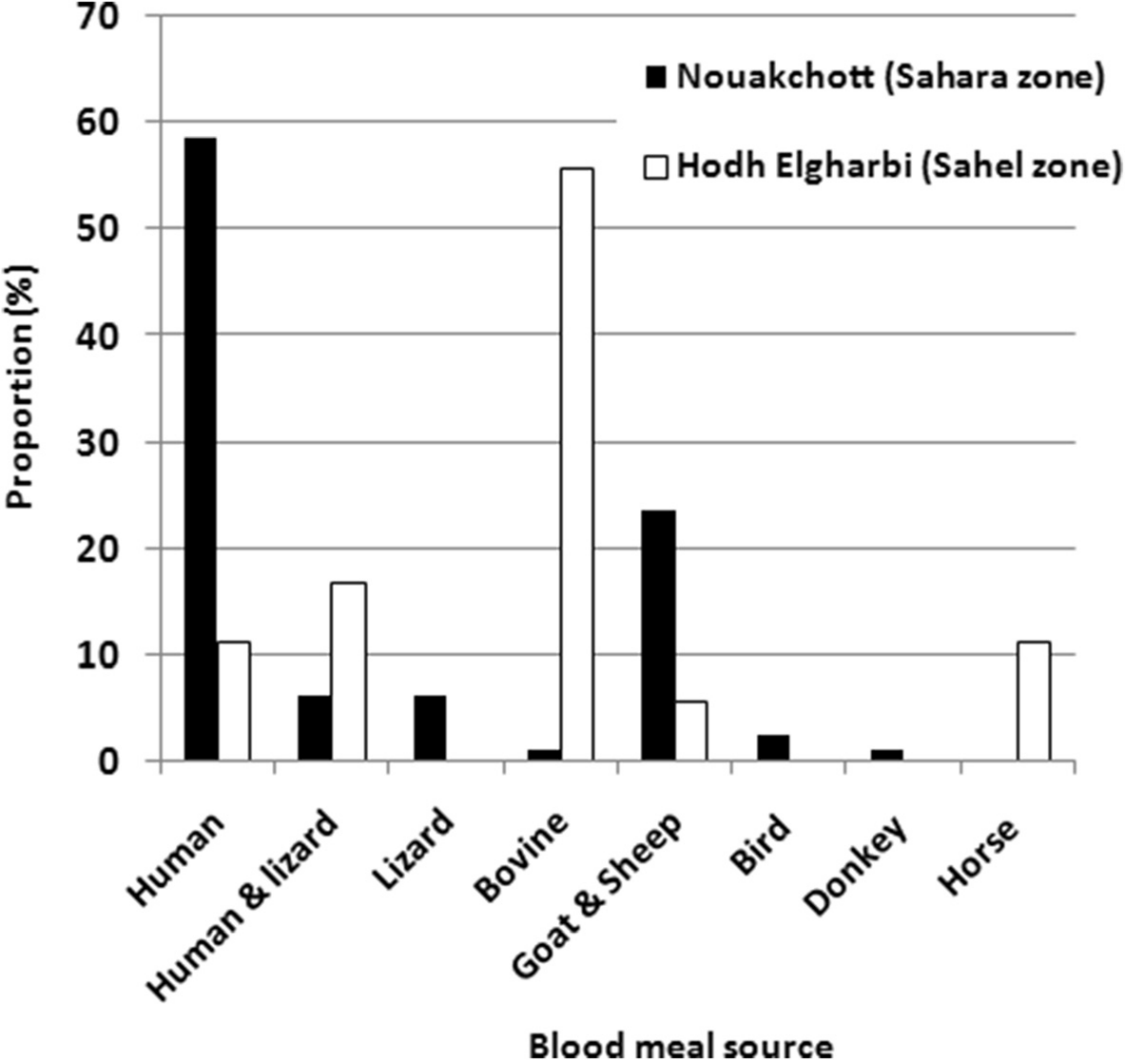
Source of blood meal of indoor resting *Anopheles arabiensis* collected using pyrethrum spray in Nouakchott region in 2009 and 2010 and in Hodh Elgharbi region in 2010

Blood-feeding patterns of resting female mosquitoes were analysed in 80 and 18 specimens of *An. arabiensis* from Nouakchott and Hodh Elgharbi regions, respectively, and the results are depicted in Fig. 3. In Nouakchott, human was the preferred single host for 58.7 % (47/80) of the female mosquitoes tested, followed by lizard with 6.2 % (5/80) of the specimens tested. Multiple host feedings (i.e. single mosquitoes taking blood from different types of hosts) were detected in 30 % (24/80) specimens. The observed combinations were sheep and goat (23.7 %; 19/80), and human and lizard (6.5 %, 5/80). In the Hodh Elgharbi region, single host feeding patterns were found in the following order: bovine hosts (55.5 %, 10/18), humans (11.1 %, 2/18) and horses (11.1 %, 2/18). There was a statistically significant difference between the percentages of human blood-fed female *An. arabiensis* in Nouakchott (58.7 %, 47/80) and in Hodh Elgharbi (11.1 %, 2/18) (Fisher’s exact test, *P* < 0.001). In Nouakchott, only three out of 186 female *An. arabiensis* tested by ELISA were found positive with CSP antigen of *P. vivax* VK210, corresponding to an overall sporozoite rate of 1.6 % (Table 2). However, PCR confirmed *P. vivax* infection in only one of these as demonstrated by the presence of a 120 bp fragment characteristic of *P. vivax*. No female anopheline mosquito was found positive with *P. falciparum* or *P. vivax* VK247. In Hodh Elgharbi, among 430 female *An. gambiae* (*s.l.*) and 87 *An. rufipes* tested, none was found positive with *Plasmodium* sporozoites.

**Table 2:** Number of circumsporozoite protein (CSP) positive mosquitoes and corresponding sporozoite rate using ELISA in *Anopheles gambiae* (*s.l.*) collected using pyrethrum spray in Nouakchott (Saharan zone) in 2009 and 2010 and in Hodh Elgharbi regions (Sahelian zone) in 2010

Study sites	No. of females tested	*Plasmodium* sp.		
		*P. vivax* VK210 (%)	*P. vivax* VK247 (%)	*P. falciparum*
Nouakchott				
Teyarett	76	1	0	0
Dar Naim	110	2	0	0
Sebkha	0	0	0	0
Total	186	3 (1.6)	0	0
Hodh Elgharbi				
Kobeni	290	0	0	0
Tintane	140	0	0	0
Total	430	0	0	0

### Prevalence of *kdr* and *ace-1* mutations

In Nouakchott, 51.7 % (75/145) of *An. arabiensis* examined for the presence of east and west African *kdr* mutations presented the wild-type allele (L1014) and 48.3 % (70/145) harboured the east-African (kdr-e) mutation (L1014S) at the homozygous status (Table 3). However, in the Hodh Elgharbi region, analysis of the *kdr* polymorphisms in 40 female *An. arabiensis* showed 80 % (32/40) with wild-type, 10 % (4/40) with west African (kdr-w) mutation and 10 % (4/40) harbouring the east-African (kdr-e) mutation. The difference in the two ecological zones for kdr-e (L1014S) mutation was highly significant (*P* < 0.001). Nevertheless, kdr-w (L1014F) mutation was found only in *An. gambiae* populations in Hodh Elgharbi region. No *ace-1* mutation was observed in mosquito specimens from the two study zones.

**Table 3:** Results of *kdr* polymorphism in *An. gambiae* (*s.l*.) caught using pyrethrum spray in Nouakchott (Saharan zone) in 2009 and 2010 and in Hodh Elgharbi region (Sahelian zone) in 2010

Study site	No. of mosquitoes tested	No. (%) of Wild-type	No. (%) of Kdr-w	No. (%) of Kdr-e
		L1014	L1014F	L1014S
Nouakchott				
Teyarett	56	27 (48.2)	0	29 (51.8)
Dar Naim	89	48 (53.9)	0	41 (46.1)
Sebkha	0	0	0	0
Total	145	75 (51.7)	0	70 (48.3)
Hodh Elgharbi				
Kobeni	13	11 (84.6)	1 (7.7)	1 (7.7)
Tintane	27	21 (77.8)	3 (11.1)	3 (11.1)
Total	40	32 (80.0)	4 (10.0)	4 (10.0)

## Discussion

We chose to use PSC as mosquito sampling method. The reference method to sample anopheline mosquitoes and determine their feeding behaviour is the use of human landing catches (HLC). Nevertheless, this method is time-consuming, expensive, sometimes difficult to implement due to logistic constraints, does not allow a study on the resting behaviours, and is unethical if it exposes human subjects to bites of infected mosquitoes [23]. In itself, the study of the resting behaviour of *Anopheles* spp. mosquitoes is also a challenge mostly concerning the exophilic behaviour and, most of the time, is not conducted as it necessitates the construction of specific structures, such as e.g. experimental huts. PSC is one of the alternative methods commonly used for collecting mosquitoes resting indoors [24–26]. This method is fast and simple, and does not require extensive skills. In addition, it allows the collection of a large number of mosquitoes of varying physiological conditions, provides valuable information on relative changes in seasonal abundance of endophilic vectors, and captures adults for determining sporozoite rates and information on host preferences and degree of exophily.

The present study provides current information on the distribution of five *Anopheles* spp., namely *An. arabiensis*, *An. rufipes*, *An. gambiae*, *An. pharoensis* and *An. funestus*, their feeding habits, and the distribution and prevalence of the knockdown resistance (*kdr*) and acetylcholine esterase (*ace-1*) mutations in two ecological zones of Mauritania. *Anopheles arabiensis* was found in both the Saharan zone (Nouakchott) and the Sahelian zone (Hodh Elgharbi), whereas the presence of the other *Anopheles* spp., including *An. gambiae* and *An. funestus*, was restricted to the Sahelian zone. This distribution pattern is in agreement with that reported by Dia et al. [7] with the same sampling method in five Sahelian regions of Mauritania including Hodh Elgharbi, except that our study also reported for the first time the presence of *An. rufipes* in the Hodh Elgharbi region. In a recent study conducted in Senegal, *An. rufipes* was frequently found in villages located in wooded and shrubby savanna [26].

Contrary to the other species, *An. arabiensis* can endure drought and is also adapted to temporary or permanent breeding sites, and its presence both in the “humid” sahelian zone and in the dry Saharan zone is not surprising. Similarly, the breeding sites of *An. funestus*, *An. rufipes* and *An. gambiae* necessitate continuous pockets of water that persist for several months during the dry season like in the Sahelian zone and their absence in the Saharan zone is congruent with their biology [27]. *Anopheles rufipes* is a widespread and commonly occurring mosquito in West Africa outside of forested areas and its presence in Mauritania has already been reported [14]. This species is predominantly zoophilic and its density could be very high in the presence of abundant cattle. Hodh Elgharbi is situated in the east Sahelian zone that is the most important herding area and accounts for 64 % of cattle, 49 % of sheep and goat population and 40 % of the camel herd of Mauritania. The abundance of *An. rufipes* in Hodh Elgharbi is congruent with the historical data, its biology and the presence of abundant potential sources of blood meals. The absence of *An. rufipes* reported by Dia et al. [7] could be explained by a lack of power but also possibly by the presence of exophilic populations that are resting in outdoor haunts such as crevices, pits and banks as described in East and southern Africa [14].

The results of host-feeding patterns suggest that considerable numbers of *An. gambiae* (*s.l.*) in Hodh Elgharbi were found to be more zoophilic than anthropophilic, compared to those of Nouakchott where tested mosquitoes equally fed on animal and human hosts. Many factors such as human host availability in each region (the population density in Nouakchott is 2.8-fold higher than in Hodh Elgharbi and the prevalence of blood meal was 4.4-fold higher) could explain the marked difference in blood meal patterns among *An. arabiensis* mosquitoes in the two regions including different resting or feeding behaviours. According to the Ministry of Livestock of Mauritania, 65 % of small ruminants (goats and sheep) of Mauritania are slaughtered in Nouakchott, mainly for leather industry. Nouakchott is therefore concentrating a large proportion of the small ruminant livestock. This, together with feeding preferences, could explain the important role of sheep and goats as sources of blood meals for *An. arabiensis* in Nouakchott compared to Hodh Elgharbi.

It is worth noting that *An. arabiensis* has been described as a zoophilic, exophagic and exophilic species [28] and also has been known to have a wide range of feeding and resting patterns, depending on geographical location [29, 30]. This behavioural plasticity allows *An. arabiensis* to adapt quickly to counter indoor residual spraying control, where suitable genotypes occur [31]. Thus, it could be assumed that, at least in Nouakchott, this malaria vector has similar exophagic and endophagic behaviours. The exophagic behaviour of *An. arabiensis* was frequently observed in areas of unstable malaria [30]. Due to their observed exophagic preference, the probability that *An. arabiensis* bites an animal host is higher than a human host, particularly in rural areas, which explains their commonly reported zoophily [32]. Therefore, their zoophilic behaviour in Hodh Elgharbi is not surprising since the region is mostly rural.

Three specimens of *An. arabiensis* from Nouakchott were detected positive for *P. vivax* VK 210 infections (CSP rate of 1.6 %) using ELISA but only one was confirmed by PCR. False positive circumsporozoite ELISA is not uncommon, especially when dealing with zoophilic species. This phenomenon has been described for *P. vivax* and *P. falciparum* and is attributed to cross-reacting antigen. False positive CSP-ELISA results are usually associated with zoophilic mosquito species but also with unidentified factors present in the bovine blood or pig blood [33–35]. That is why it is highly recommended to confirm all positive CSP-ELISA results, by performing *Plasmodium*-specific PCR, followed, if possible by sequencing of the amplicons for *Plasmodium* species determination when dealing with zoophilic vectors and before vector incrimination. False positive CSP-ELISA is a challenge for the estimation of the entomological inoculation rate of malaria and for vector incrimination.

The presence of *An. arabiensis* in Nouakchott and its infection confirmed by CSP and PCR provide a strong evidence that this species is likely to play an important role in malaria transmission, which has been recently established in the capital city [2, 3]. In southern Mauritania, the infection rate of *An. gambiae* (*s.l*.) has been previously estimated at 0.17 % (one infected specimen on 594) [7]. In Hodh Elgharbi, our results are similar to those previously published by Dia et al. [7] in southern Mauritania. The infection rates are not statistically different between the two studies and the absence of infected *Anopheles* spp. in our study could be due only to a lack of power. Our results are compatible with an infection rate of 0.17 % in the area as previously described. In the Hodh Elgharbi region, despite the presence of three efficient malaria vectors (*An. gambiae*, *An. arabiensis* and *An. funestus*) among collected anopheline species, the absence of infected mosquitoes in the present study and in the study conducted by Dia et al. [7] contrasts with the observed malaria burden in the region [1, 36, 37]. This could be the result of the small sample size of engorged anopheline specimens captured in this region but also due to the sampling method used in the two studies that allowed only sampling indoor resting *Anopheles* spp. If the major part of the population (including the anthropophagic specimens) is exophilic, the roles of *An. gambiae*, *An. arabiensis* and *An. funestus* in malaria transmission could be totally underestimated by PSC. The discrepancy between the burden of malaria in Hodh Elgharbi and the absence of infected *Anopheles* spp. could be explained by a sampling bias.

The results of the present study also provide for the first time information on two important genes involved in insecticide resistance in the studied anopheline populations from the two regions. Although wild-type *kdr* was predominant among *An. arabiensis* in the two regions, both *kdr* point mutations (L1014S and L1014F) were found to co-exist in the mosquitoes examined. The most important finding in this context was the presence of a high proportion of the east African mutation (L1014S) among *An. arabiensis* from Nouakchott. Indeed, L1014S allele has never been reported west of 10°W in Africa. This point mutation, which initially predominated in east Africa [38], has recently been reported in west Africa, first in Benin [39], then in Burkina Faso [11] and Senegal [12] in *An. arabiensis* populations. Further studies are needed to elucidate the origin of this mutation in Nouakchott. The role of *An. funestus* in the Hodh Elgharbi region should be further examined using other complementary methods, such as human landing catches and light traps, to arrive at a more definite conclusion on the potential of this mosquito as a malaria vector in this region. Moreover, because of the relatively high proportions observed for *An. rufipes* in Hodh Elgharbi (394 specimens from Kobeni and 184 specimens from Tintane), the determination of the role of this species in malaria transmission needs further investigation to incriminate it. A recent study in Burkina Faso has suggested the involvement of *An. rufipes* in malaria transmission as an oocyst has been found in one specimen [40]; this is not the first time that this situation has been described. *Anopheles rufipes* mosquitoes harboring oocysts or sporozoites have been sporadically caught in various countries in west and south Africa while at the same time no infection was retrieved in a large sample of 4000 specimens. The most obvious explanation for all these conflicting findings seems to be that the sporozoites observed were of non-human origin, a conclusion compatible with the sporadic nature of positive findings. As no molecular investigation has yet been conducted on *An. rufipes* specimens harbouring oocysts or sporozoites, its implication in malaria transmission remains unclear.

Insecticide resistance surveys should also be undertaken to correlate *kdr* genotypes to DDT and pyrethroid resistance phenotype. This would allow ascertaining the phenotypic effect of both *kdr* mutations, particularly when co-occurring in the same individual. Furthermore, the presence of other mechanisms of resistance could also be highlighted by the World Health Organisation susceptibility tests.

## Conclusion

Although the present study tested a limited number of *Anopheles* spp. mosquitoes from a limited area using only one capture method with a limitation of not sampling the exophilic species, the results clearly incriminate *An. arabiensis* as the major malaria vector in Nouakchott. We also described for the first time the absence of *ace-1* mutation, the co-occurence of both west and east African *kdr* mutation in *An. gambiae* in Mauritania, and highlighted the regional variations in the prevalence and type of *kdr* mutations. Complementary entomological investigations (human landing catches, light traps, MM traps and/or experimental huts or tents) are necessary to identify the malaria vectors in the area and to propose adapted vector control measures.
